# Immune Checkpoint Inhibitor Combinations in Advanced Ovarian Cancer: A Systematic Review and Meta-Analysis of Randomized Controlled Trials

**DOI:** 10.3390/cancers18182971

**Published:** 2026-09-14

**Authors:** Boram Choi, Youn Jin Choi, Keun Ho Lee

**Affiliations:** Department of Obstetrics and Gynecology, Seoul St. Mary’s Hospital, College of Medicine, The Catholic University of Korea, Seoul 06591, Republic of Korea

**Keywords:** ovarian cancer, immune checkpoint inhibitor, PD-1, PD-L1, PARP inhibitor, bevacizumab, meta-analysis, systematic review, combination therapy

## Abstract

Background/Objectives: Immune checkpoint inhibitors (ICIs) have shown limited efficacy in the treatment of ovarian cancer when used alone. This systematic review and meta-analysis evaluated the impact of different combination partners on ICI efficacy. Methods: PubMed, Embase, CENTRAL, and Web of Science were searched until March 2026 for phase III randomized controlled trials (RCTs) of anti-PD-1/PD-L1 combination regimens. Nine fully published trials (*N* = 7381) were included in the primary analyses; an ICI monotherapy trial and two conference-abstract-only trials were considered in sensitivity analyses. Hazard ratios (HRs) were pooled using random-effects models with pre-specified subgroup analyses by combination partner. Evidence certainty was assessed using GRADE. Results: Overall, ICI combination therapy significantly improved progression-free survival (PFS) (HR 0.83, 95% confidence interval (CI) 0.73–0.93; *I*^2^ = 55.8%; *p* = 0.007). Bevacizumab plus ICI significantly improved progression-free survival (PFS) (*k* = 4; HR 0.83, 95% CI 0.74–0.93 [Wald-type]; GRADE: Moderate). PARP inhibitor plus ICI also showed a Wald-type PFS improvement (*k* = 3; HR 0.78, 95% CI 0.63–0.96 [Wald-type]; GRADE: Low), but this was not robust to Hartung–Knapp–Sidik–Jonkman (HKSJ) adjustment or inclusion of abstract-only trials (SA5: *k* = 5; HR 0.85, 95% CI 0.67–1.09; *I*^2^ = 90.1%). Chemotherapy plus ICI (two avelumab trials) showed no benefit (*k* = 2; HR 0.96, 95% CI 0.66–1.38). KEYNOTE-B96 demonstrated the first significant overall survival (OS) benefit with an ICI regimen (HR 0.82, 95% CI 0.69–0.97). Funnel plot assessment showed no asymmetry; Egger’s test was not formally applied, as the pre-specified threshold of ten trials was not met (descriptive *p* = 0.81). Conclusions: ICI combination therapy improved PFS in advanced ovarian cancer, in contrast to earlier syntheses pooling monotherapy and combination regimens. The benefit was most consistent for bevacizumab-based regimens (GRADE: Moderate); the PARP inhibitor–based benefit was less robust (Low), and chemotherapy combinations offered no advantage (Very low). Subgroup differences were not statistically significant and remain exploratory; a partner-specific, biomarker-guided approach is warranted.

## Simple Summary

Immune checkpoint inhibitors (ICIs) are a promising cancer treatment; however, their effectiveness in ovarian cancer is limited when used alone. This study systematically reviewed nine phase III clinical trials involving 7381 patients to understand the performance of ICIs when combined with other therapies, such as PARP inhibitors, bevacizumab, or chemotherapy. Overall, ICI combination therapy reduced the risk of disease progression or death by approximately 17%. Our findings further suggest that the choice of combination partner influences the robustness of this benefit. Specifically, the combination of ICIs with bevacizumab showed a consistent benefit in slowing cancer progression, with moderate certainty of evidence. Combinations with PARP inhibitors also showed benefits, but with lower certainty, often depending on specific trial designs. However, ICIs combined with chemotherapy did not show any significant improvement. This highlights the importance of choosing the appropriate combination therapy and tailoring treatment to individual patients for better outcomes in ovarian cancer.

## 1. Introduction

Epithelial ovarian cancer (EOC) is the most lethal gynecological malignancy, responsible for 314,000 new diagnoses and 207,000 deaths worldwide annually [1]. Surgery and platinum-based chemotherapy have improved outcomes; nevertheless, the majority of women with advanced disease experience relapse within three years, and fewer than half survive beyond five years [2].

Immune checkpoint inhibitors (ICIs) directed against programmed cell death protein 1 (PD-1) and its ligand PD-L1 have reshaped the treatment of melanoma, non-small cell lung cancer, and renal cell carcinoma [3]. Ovarian cancer has proven to be a stubborn exception, with single-agent response rates of only 8–15% [4,5]. This resistance is typically ascribed to an immunologically “cold” tumor microenvironment, characterized by a low tumor mutational burden and limited infiltration [6].

This has motivated a series of phase III randomized controlled trials (RCTs) pairing ICIs with agents such as PARP inhibitors, bevacizumab, and platinum-based chemotherapy. Each pairing rests on a distinct mechanistic argument: PARP inhibitors are hypothesized to accumulate DNA damage and increase neoantigen presentation [7], bevacizumab may normalize tumor vasculature and improve immune cell infiltration [8], and cytotoxic chemotherapy can provoke immunogenic cell death [9]. However, direct evidence for these mechanisms in specific ovarian cancer populations and regimens remains limited.

A recent meta-analysis by Vida et al. pooled 10 RCTs and found no overall progression-free survival (PFS) benefit for anti-PD-1/PD-L1 therapy in ovarian cancer (hazard ratio (HR) 0.98, 95% confidence interval (CI): 0.85–1.12) [10]. This contrasts with prior reviews; although the analysis encompassed immune checkpoint inhibitors (ICIs) as a unified category, it did not explore the significance of specific combination partners. Distinguishing between PARP inhibitor-, bevacizumab-, and chemotherapy-based pairings is clinically important, as each combination may have distinct efficacy and toxicity profiles.

The evidence base shifted again with KEYNOTE-B96 (ENGOT-ov65), reported in The Lancet in 2026 [11]. In platinum-resistant disease, adding pembrolizumab to weekly paclitaxel, with or without bevacizumab, improved both PFS and overall survival (OS). This was the first randomized signal of an OS gain from an ICI regimen in ovarian cancer, and it postdated earlier meta-analysis results.

Therefore, we conducted this systematic review and meta-analysis with three aims: to weigh the overall efficacy and safety of ICI combinations in advanced ovarian cancer; to conduct pre-specified subgroup analyses comparing their effects across PARP inhibitor-, bevacizumab-, and chemotherapy-based regimens (a partner-stratified comparison that, to our knowledge, has not been the focus of prior pairwise meta-analyses of ICI therapy in ovarian cancer [10]); and to grade the certainty of evidence using the GRADE framework.

## 2. Methods

### 2.1. Protocol and Registration

This systematic review was conducted and reported following the Preferred Reporting Items for Systematic Reviews and Meta-Analyses (PRISMA) 2020 guidelines [12]; the completed PRISMA 2020 checklist is provided in the Supplementary Materials. The protocol was prospectively registered with the PROSPERO database (CRD420261336510).

### 2.2. Search Strategy

**Figure 1:**
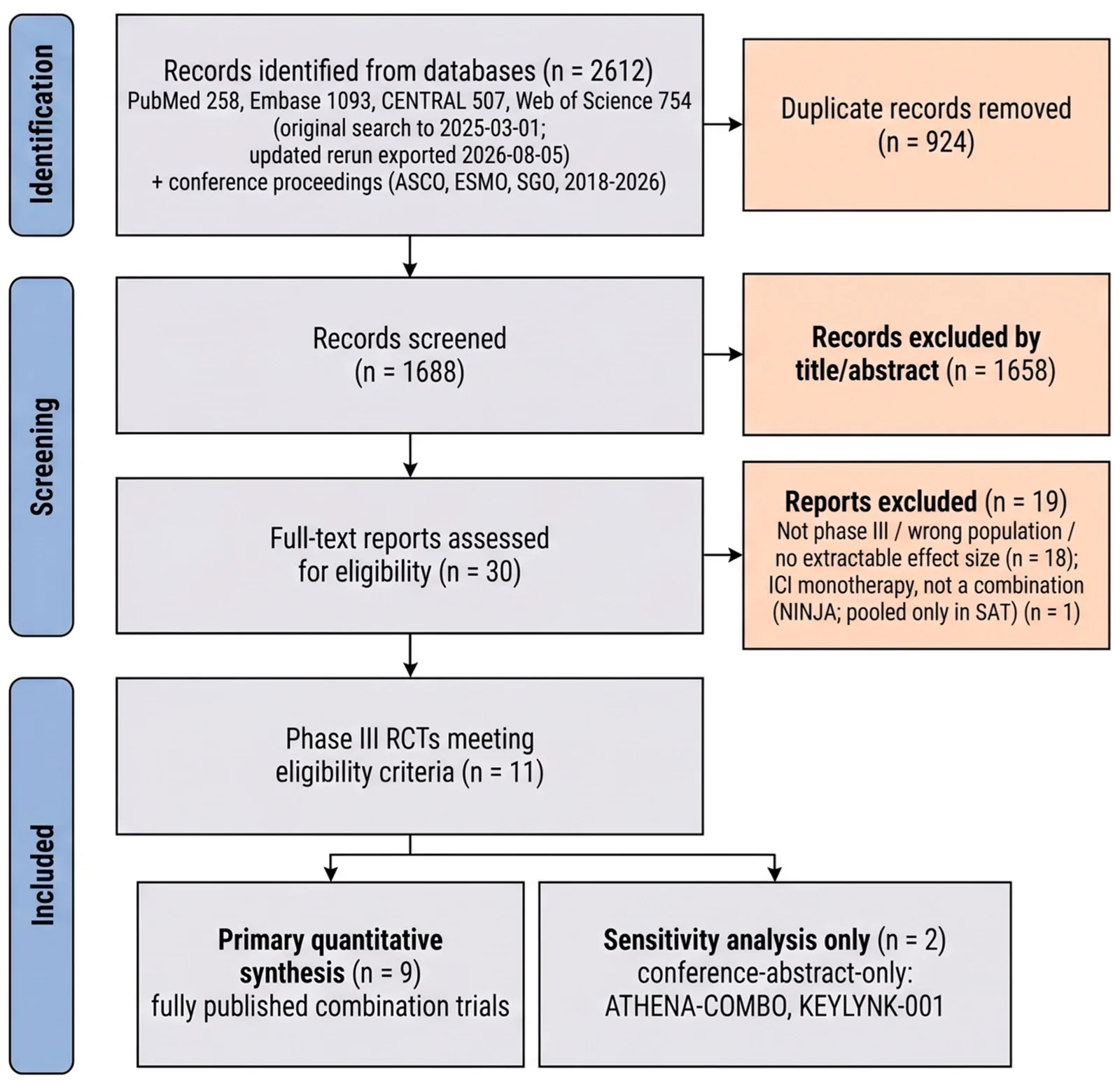
PRISMA 2020 flow diagram of study selection. Identification and duplicate removal counts were generated from the 5 August 2026 updated rerun export of all four databases (union-find on DOI, PMID, Embase PUI, and normalized title; supplemented with DOIs excluded). Full-text stage counts and the 11 included trials are unchanged from the primary screening. ICI monotherapy was excluded at full-text review and pooled only in sensitivity analysis SA1.

A comprehensive literature search was performed across four electronic databases: PubMed/MEDLINE, Embase (via Ovid), Cochrane Central Register of Controlled Trials (CENTRAL), and Web of Science (Science Citation Index) from inception to 1 March 2026. Records identified by this original search formed the basis of study selection. Conference proceedings of the ASCO, ESMO, and SGO annual meetings (2018–2026) were manually searched as part of the original search. To generate fully documented, reproducible identification and deduplication counts for reporting, all four database searches were rerun and re-exported on 5 August 2026; this updated rerun served a documentation purpose only: the March and August result sets were not combined, the rerun did not replace the original search, and no record identified in the rerun altered the eligibility decisions of the original search. Cross-database deduplication was performed by matching DOI, PMID, Embase identifier, and normalized title (Figure 1). The conference-proceedings search was not rerun in August 2026.

The search strategy combined terms related to (1) ovarian cancer population (MeSH terms and free text), (2) immune checkpoint inhibitors (specific drug names and class terms), (3) combination partners (PARP inhibitors, bevacizumab, and chemotherapy agents), and (4) study design (randomized controlled trials). The complete search strategy is presented in Supplementary Table S1.

The reference lists of the included studies and relevant systematic reviews were manually searched for additional eligible studies.

### 2.3. Eligibility Criteria

Inclusion criteria:

- Population: Adult women (≥18 years) with advanced (FIGO Stage III–IV) or recurrent epithelial ovarian, fallopian tube, or primary peritoneal cancer.
- Intervention: Anti-PD-1 or anti-PD-L1 ICI-containing combination regimens (pembrolizumab, nivolumab, atezolizumab, avelumab, durvalumab, or dostarlimab combined with at least one non-ICI partner: a PARP inhibitor, bevacizumab, and/or chemotherapy). Pure ICI monotherapy trials were not eligible; the single monotherapy trial identified (NINJA [13]) was pooled only in a pre-specified sensitivity analysis to contextualize the estimates against prior syntheses that included it (SA1, Section 2.6).
- Comparator: Standard treatment without ICI (with or without placebo).
- Outcomes: PFS (primary); OS, objective response rate (ORR), and safety (secondary).
- Study design: Randomized controlled trials (Phase III).

Exclusion criteria:

- Single-arm studies without comparator.
- Phase I or dose-finding studies.
- Non-epithelial ovarian cancer.
- Duplicate publications (most recent data retained).
- Conference abstracts without full-text publication were not eligible for the primary quantitative synthesis. However, definitive phase III trials available only as conference abstracts with extractable effect sizes (e.g., ASCO, ESMO, and SGO late-breaking abstracts) were considered eligible for the review and were included in a pre-specified sensitivity analysis (SA5/SA-NRG), while remaining excluded from the primary analysis because of the limited reporting completeness and non–peer-reviewed status of abstract-only data.
- Anti-CTLA-4 monotherapy studies.

### 2.4. Study Selection and Data Extraction

Two reviewers (B.C. and Y.J.C.) independently screened the titles and abstracts, followed by full-text screening. Disagreements were resolved by consensus or consultation with a third reviewer (K.H.L.).

Data were extracted using a standardized form, including study characteristics (trial name, phase, sample size, blinding, and setting), patient demographics (age, FIGO stage, and histology), biomarker status (BRCA, HRD, and PD-L1), intervention details (ICI agent, combination partner, and dosing schedule), and outcomes (PFS HR with 95% CI, OS HR, ORR, grade ≥ 3 adverse events, and immune-related adverse events).

When multiple publications from the same trial were available, the most complete and definitive publication was used for each outcome (e.g., the primary ATALANTE publication [14] for PFS and the final-analysis publication [15] for the overall survival narrative; the ATHENA program design paper [16] and the ATHENA-COMBO results abstract [17]; the NRG-GY009 primary-results abstract [18] and its companion biomarker analysis [19]). Data for the same outcome were not extracted from more than one report, to avoid double-counting.

### 2.5. Risk of Bias Assessment

The risk of bias for each included trial was assessed using the Cochrane Risk of Bias 2.0 (RoB 2) tool [20], which evaluates five domains: randomization process, deviations from intended interventions, missing outcome data, outcome measurement, and selection of reported results. Each domain was rated as having a low risk of bias, some concerns, or a high risk of bias.

### 2.6. Statistical Analysis

The hazard ratios (HRs) and their corresponding 95% confidence intervals (CIs) for progression-free survival (PFS) and overall survival (OS) were aggregated employing a random-effects model. (restricted maximum likelihood [REML] estimator). The Hartung–Knapp–Sidik–Jonkman (HKSJ) method was used for confidence interval adjustment of the overall pooled estimate and subgroup estimates comprising at least six studies [21]. For subgroup estimates with five or fewer studies (*k* ≤ 5), both standard Wald-type and HKSJ confidence intervals are reported: the Wald-type interval is presented as the primary estimate, and the HKSJ interval is reported alongside it to transparently convey the additional uncertainty at small *k*, where the HKSJ correction can yield substantially wider intervals [21]. Therefore, the statistical significance of small-k subgroup estimates was interpreted conservatively, with a subgroup effect regarded as robust only when it remained significant under HKSJ adjustment. Statistical heterogeneity was assessed using the *I*^2^ statistic and Cochran’s Q-test. *I*^2^ values of <25%, 25–50%, 50–75%, and >75% were considered indicative of low, moderate, substantial, and considerable heterogeneity, respectively [22].

Trials were assigned to combination-partner subgroups using an explicit hierarchical rule: (1) when the ICI-specific randomization concerned the maintenance phase, the maintenance partner defined the subgroup (ANITA: niraparib; DUO-O: olaparib); (2) otherwise, a concomitant non-chemotherapy backbone agent co-administered with the ICI defined the subgroup (bevacizumab in IMagyn050, ATALANTE, AGO-OVAR 2.29, and KEYNOTE-B96); and (3) regimens adding an ICI to chemotherapy alone were assigned to the chemotherapy + ICI subgroup (JAVELIN Ovarian 100 and 200). For the factorial ±bevacizumab design of KEYNOTE-B96, the trial was assigned to the bevacizumab + ICI subgroup because bevacizumab co-administration was part of the permitted regimen in both arms; this assignment was varied in a sensitivity analysis (SA3). Because multi-agent regimens can contain more than one partner (e.g., durvalumab + bevacizumab + olaparib in DUO-O), categories are not mutually exclusive at the regimen level; the primary assignment and its rationale are documented for each trial in Supplementary Table S8, and alternative assignments were tested in pre-specified sensitivity analyses (SA2 and SA3).

Pre-specified exploratory subgroup analyses were performed as follows:

- Combination partner: PARP inhibitor + ICI vs. bevacizumab + ICI vs. chemotherapy + ICI.
- Treatment setting: first-line vs. platinum-sensitive recurrent vs. platinum-resistant.
- ICI agent: pembrolizumab vs. atezolizumab vs. durvalumab vs. others.
- Biomarker status (BRCA, HRD, PD-L1): narrative synthesis only, as individual patient data were not available for pooled biomarker-stratified analysis. These subgroup comparisons were exploratory and hypothesis-generating. With only two to four trials per subgroup, they cannot provide robust evidence of the superiority of one partner strategy over another: tests for subgroup differences are inherently underpowered at this scale [23], no multiplicity adjustments were applied, and nominally significant subgroup estimates (particularly those with *k* ≤ 3) must be interpreted with particular caution.

The sensitivity analyses included:

- Exclusion of trials with “some concerns” for risk of bias (SA-RoB).
- Fixed-effect model comparison.
- SA1: inclusion of the NINJA trial, which compared ICI monotherapy (nivolumab) with chemotherapy and therefore did not meet the combination-therapy eligibility criterion; it was pooled in this sensitivity analysis solely to benchmark the present combination-only estimates against prior meta-analyses that included monotherapy trials [10].
- SA2: reclassification of DUO-O from the PARP inhibitor + ICI subgroup to the bevacizumab + ICI subgroup, given its triple combination design (durvalumab + bevacizumab + olaparib).
- SA3: reclassification of KEYNOTE-B96 from the bevacizumab + ICI subgroup to the chemotherapy + ICI subgroup, given its ±bevacizumab factorial design.
- Two additional phase III trials (ATHENA-COMBO [16,17] and KEYLYNK-001 [24]), available only as conference abstracts, met the eligibility criteria but were excluded from the primary analysis and included in a pre-specified sensitivity analysis (SA5) to assess their impact on the subgroup estimates.

Additionally, NRG-GY009 [18,19], reported as phase II/III conference abstracts evaluating PLD/bevacizumab/atezolizumab versus PLD/bevacizumab in platinum-resistant ovarian cancer, was also excluded from the primary analysis due to a lack of full-text publication but included in a sensitivity analysis (SA-NRG) to assess its potential impact. As NRG-GY009 reported a one-sided 99.99% CI (HR 0.79, 0.0–1.21), the standard error was back-calculated from the log-transformed upper confidence limit using the formula SE = [log(upper CI) − log (HR)]/z(0.9999), where z(0.9999) = 3.7190. A conventional 95% two-sided CI (0.63–0.99) was then derived, which, by standard alpha = 0.05 criteria, suggested significance. However, the original abstract explicitly concluded that the results were “not statistically significant,” emphasizing the need to respect the trial’s pre-specified statistical plan and highly conservative alpha level for its primary endpoints.

To evaluate publication bias, funnel plots were visually inspected, and Egger’s linear regression test was applied when there were 10 or more studies available [25]. Given the limited statistical power of Egger’s test when the number of studies is small, a non-significant result—or the inability to apply the test at all—was not interpreted as evidence that publication bias is absent [25].

The certainty of the evidence for each outcome was evaluated using the GRADE framework [26]. Two reviewers (B.C. and Y.J.C.) rated each outcome independently, with disagreements resolved by consensus or consultation with a third reviewer (K.H.L.). Randomized-trial evidence started at a high certainty rating and was downgraded, as appropriate, for risk of bias, inconsistency, imprecision, indirectness, and publication bias. Imprecision assessments explicitly accounted for the small number of trials and the width of the confidence intervals, and indirectness assessments considered differences in treatment settings (first-line, platinum-sensitive recurrent, and platinum-resistant), combination partners, and control regimens across the pooled trials. The per-outcome evidence profile, including the specific domains downgraded for each outcome, is provided in Supplementary Table S7.

For trials reporting adjusted confidence intervals (e.g., repeated CIs from interim analyses with alpha-spending), the standard error was back-calculated from the width of the reported CI bounds on the log scale using SE = [log(upper CI) − log(lower CI)]/(2 × z), where z corresponds to the reported confidence. This approach assumes a log-normal distribution of the hazard ratio and uses both bounds symmetrically to mitigate potential asymmetry from alpha spending adjustments.

All analyses were performed using R version 4.4.3 (R Foundation for Statistical Computing) with the “metafor” package (version 5.0-1).

Statistical analyses were performed by the authors with the assistance of ChatGPT (GPT-5.6) and Anthropic Claude (Opus 4.8) for code drafting and figure generation. The authors independently reviewed and verified all AI-assisted outputs against the original data sources and take full responsibility for the accuracy of the analyses.

### 2.7. Deviations from Protocol

During screening, non-English publications were excluded; however, no eligible non-English trials were identified in the databases searched, and the potential for language bias cannot be fully excluded. No other deviations from the registered protocol occurred. The handling of the monotherapy trial NINJA (not eligible per the intervention criteria; pooled only in SA1) and of conference-abstract-only trials (eligible but pooled only in SA5/SA-NRG) is described in Section 2.3 and Section 2.6.

## 3. Results

### 3.1. Study Selection

The updated rerun of the four database searches (5 August 2026; documenting the original search covering inception to 1 March 2026) identified 2612 records; after the removal of 924 duplicates, 1688 records were screened. After title/abstract screening, 30 full-text articles were assessed for eligibility. Eleven phase III randomized controlled trials (RCTs) met the eligibility criteria (Figure 1): nine fully published combination-therapy trials (*N* = 7381), which constituted the primary quantitative synthesis, and two trials available only as conference abstracts (ATHENA-COMBO [16,17] and KEYLYNK-001 [24]), which were eligible but reserved for the pre-specified sensitivity analysis (SA5). The NINJA trial [13] was excluded at full-text review because it evaluated ICI monotherapy (nivolumab versus chemotherapy) rather than an ICI-containing combination regimen; it was pooled only in SA1, for comparability with prior meta-analyses (Figure 1).

### 3.2. Study Characteristics

A total of 7381 patients with advanced or recurrent epithelial ovarian cancer (EOC) were enrolled in the nine primary trials. Four trials evaluated first-line treatment (IMagyn050 [27], JAVELIN Ovarian 100 [28], DUO-O [29], and FIRST [30]), two focused on platinum-sensitive recurrence (ATALANTE [14] and ANITA [31]), and three investigated platinum-resistant disease (JAVELIN Ovarian 200 [32], KEYNOTE-B96 [11], which was included as a fully published phase III trial (*N* = 643) with primary endpoint data (PFS HR with 95% CI) obtained from a peer-reviewed publication in The Lancet, and AGO-OVAR 2.29 [33]). The immune checkpoint inhibitors included atezolizumab (*n* = 4), avelumab (*n* = 2), durvalumab (*n* = 1), dostarlimab (*n* = 1), and pembrolizumab (*n* = 1). Six trials were double-blind, and three were open-label (Table 1). Three trials combined ICIs with PARP inhibitor-based regimens (DUO-O, FIRST, and ANITA), four with bevacizumab-based regimens (IMagyn050, ATALANTE, KEYNOTE-B96, and AGO-OVAR 2.29), and two with chemotherapy without bevacizumab (JAVELIN Ovarian 100 and JAVELIN Ovarian 200). The two abstract-only trials eligible for SA5 (ATHENA-COMBO and KEYLYNK-001) were both first-line PARP inhibitor + ICI regimens.

### 3.3. Risk of Bias

**Figure 2:**
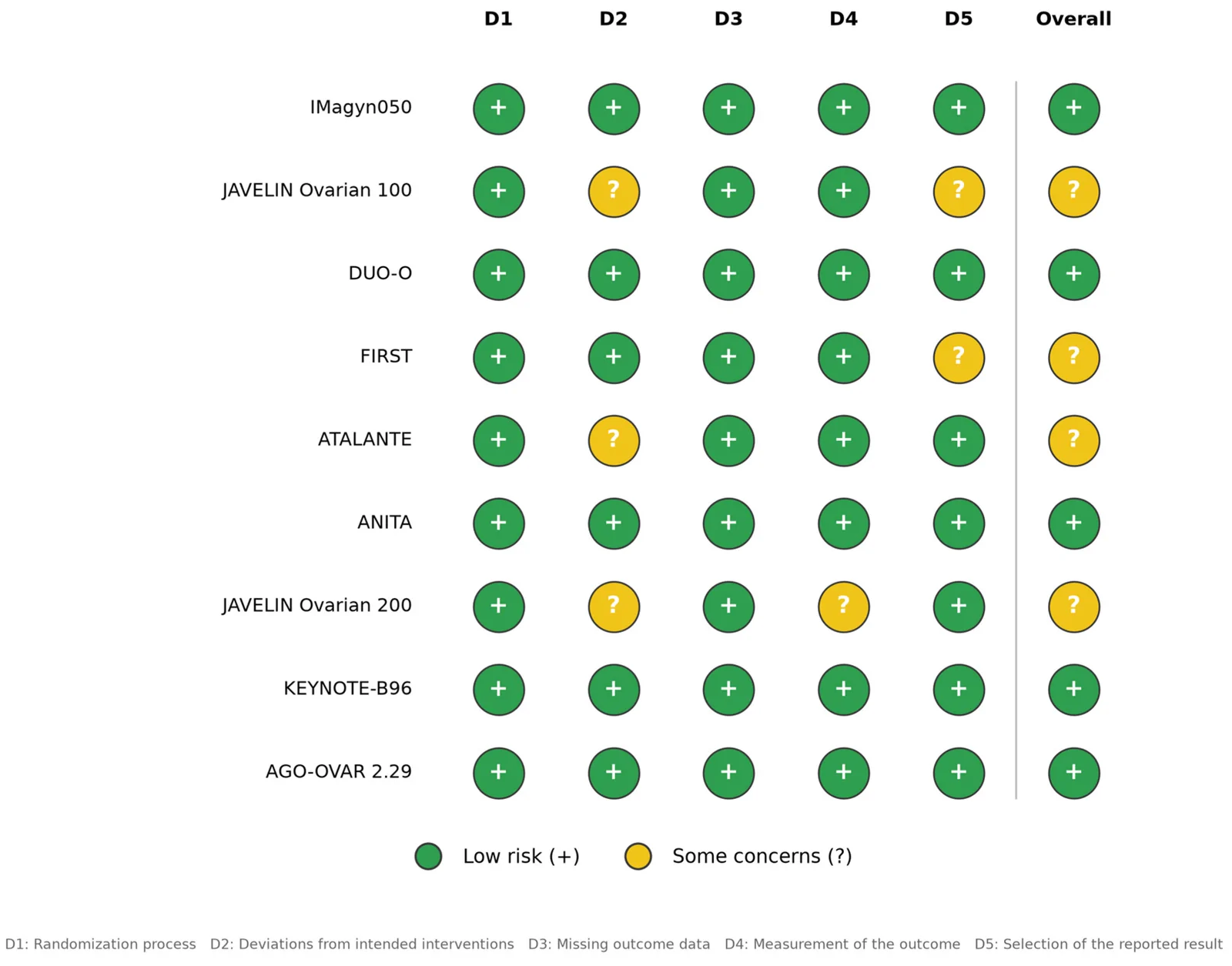
Risk of bias traffic light plot (RoB 2.0) for the nine primary analysis trials.

Among the nine primary analysis trials, five (56%) were judged to have a low risk of bias, whereas four (44%) were associated with some concerns (Table 2; Figure 2). These concerns primarily involved open-label designs (D2: JAVELIN Ovarian 100, ATALANTE, and JAVELIN Ovarian 200), investigator-assessed PFS without a blinded independent central review (D4: JAVELIN Ovarian 200), early termination for futility (D5: JAVELIN Ovarian 100), and interim analysis reporting (D5: FIRST). No trial was classified as having a high risk of bias. The monotherapy trial NINJA, pooled only in SA1, was rated as having some concerns (open-label design [D2]; investigator-assessed PFS without BICR [D4]).

### 3.4. Overall Efficacy: PFS

**Figure 3:**
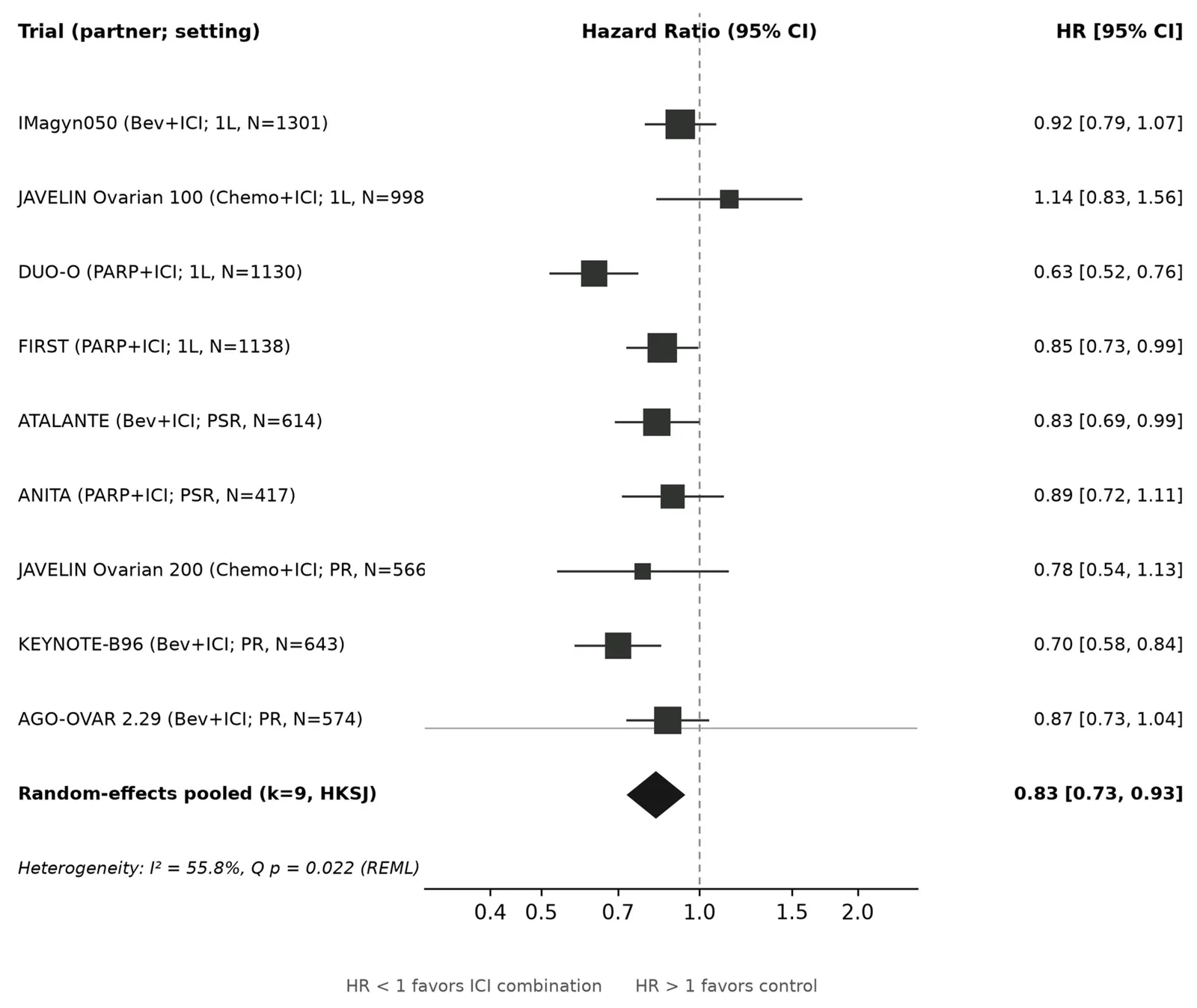
Forest plot of pooled hazard ratios for progression-free survival (PFS) across the nine included trials. HR < 1 favors the immune checkpoint inhibitor combination. Diamonds represent pooled estimates from the random-effects model.

Across the nine primary analysis trials, immune checkpoint inhibitor (ICI) combination therapy significantly improved progression-free survival (PFS) (hazard ratio [HR] 0.83, 95% confidence interval [CI] 0.73–0.93; *p* = 0.007; 95% prediction interval [PI] 0.62–1.10; Figure 3), with moderate heterogeneity (*I*^2^ = 55.8%; Cochran’s Q *p* = 0.022).

### 3.5. Subgroup Analysis by Combination Partner

**Figure 4:**
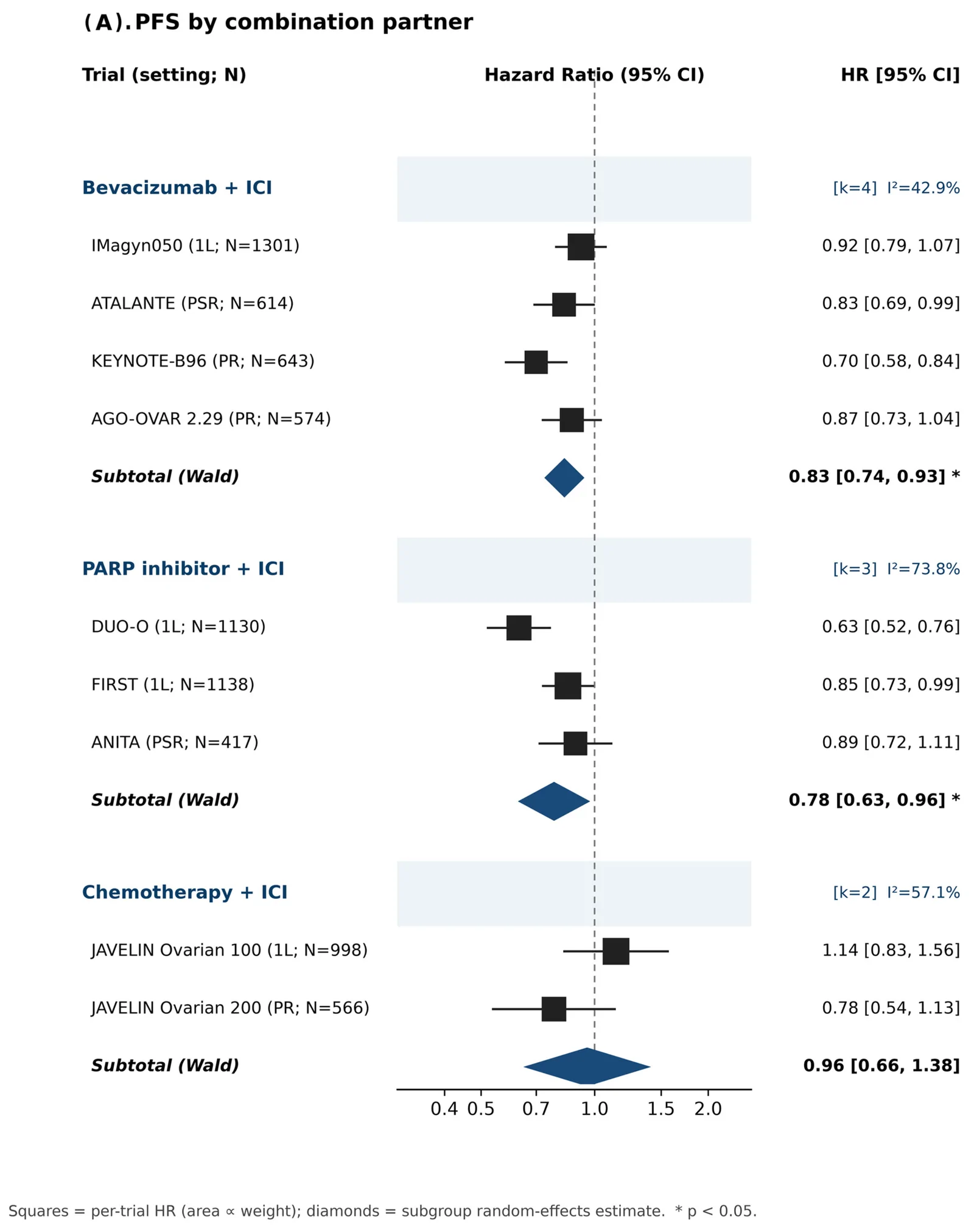

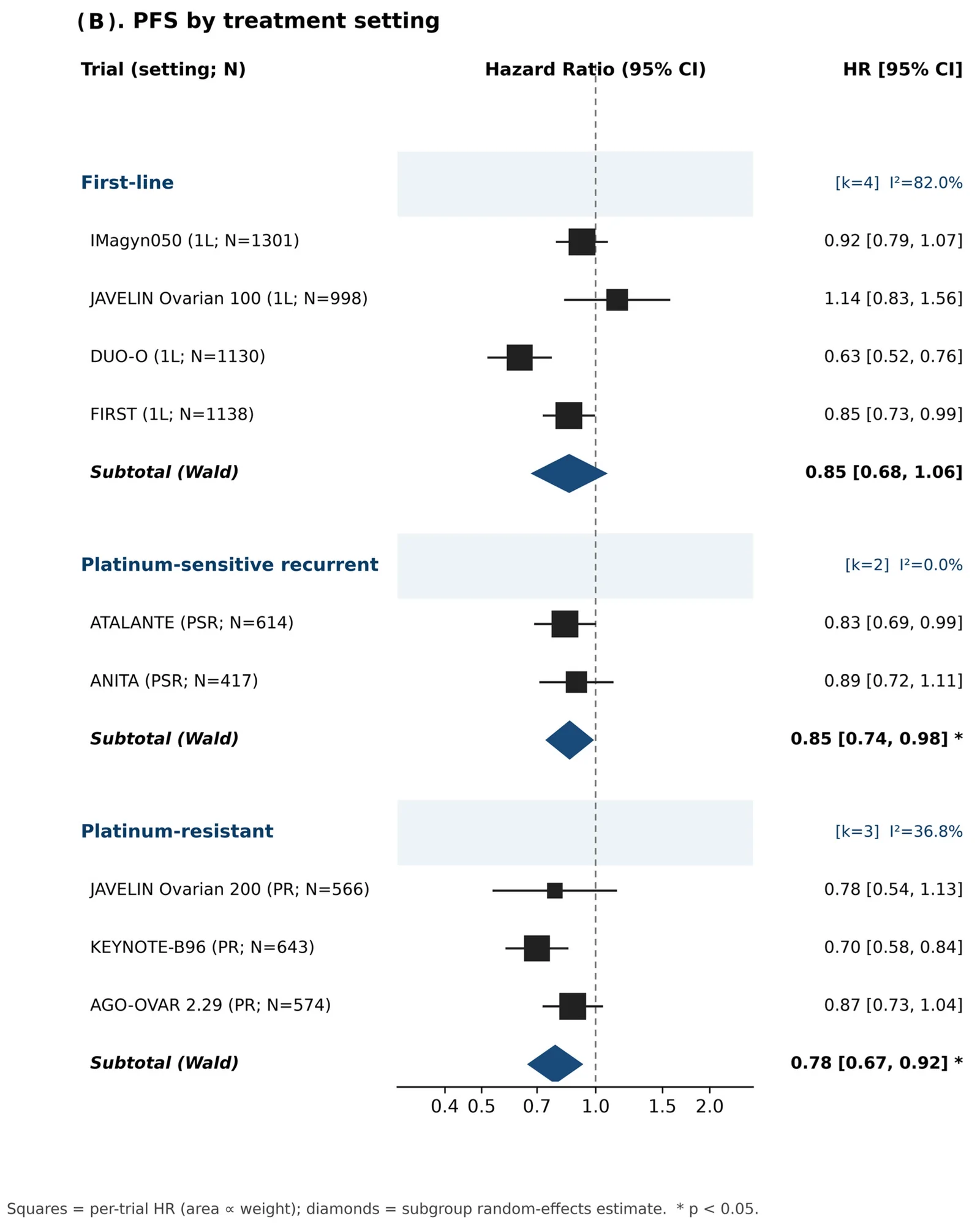
Forest plots of progression-free survival subgroup analyses. (**A**) Stratified by combination partner: bevacizumab + ICI, PARP inhibitor + ICI, and chemotherapy + ICI. (**B**) Stratified by treatment setting: first-line, platinum-sensitive recurrent, and platinum-resistant settings. The subgroup pooled estimates are represented by diamonds.

Broken down by combination of partners, treatment effects differed in clinically meaningful ways (Figure 4). Bevacizumab + ICI (*k* = 4; *N* = 3132): the addition of an ICI to bevacizumab-based regimens improved PFS with a Wald-type estimate that was significant (HR 0.83, 95% CI 0.74–0.93 [Wald-type], *p* = 0.002; *I*^2^ = 42.9%), although the HKSJ-adjusted interval was borderline and included the null (95% CI 0.69–1.00, *p* = 0.051). This benefit was directionally consistent across treatment settings, including first-line therapy (IMagyn050, HR 0.92), platinum-sensitive recurrence (ATALANTE, HR 0.83), and platinum-resistant disease (KEYNOTE-B96, HR 0.70; AGO-OVAR 2.29, HR 0.87), and it remained significant under HKSJ adjustment when DUO-O was included in this subgroup (*k* = 5; HR 0.79, 95% CI 0.65–0.96, *p* = 0.027; Section 3.12).

PARP inhibitor combined with ICI (*k* = 3; *N* = 2685): among fully published trials, the Wald-type estimate for adding an ICI to PARP inhibitor-based regimens indicated improved progression-free survival (PFS) (HR 0.78, 95% CI 0.63–0.96 [Wald-type], *p* = 0.020; *I*^2^ = 73.8%); however, this significance was not retained under the HKSJ adjustment (95% CI 0.49–1.24, *p* = 0.148). This estimate was heterogeneous, ranging from the durvalumab/bevacizumab/olaparib triplet in DUO-O (HR 0.63) through FIRST (HR 0.85) and ANITA (HR 0.89); moreover, it was not robust to the inclusion of the two abstract-only PARP inhibitor + ICI trials (Section 3.12). Chemotherapy + ICI without bevacizumab (*k* = 2; *N* = 1564; both avelumab trials): PFS did not improve (HR 0.96, 95% CI 0.66–1.38 [Wald-type]; *p* = 0.81; *I*^2^ = 57.1%); the HKSJ interval is uninformative at *k* = 2 (df = 1). The two trials pointed in opposite directions: a modest signal in JAVELIN Ovarian 200 (HR 0.78) against a detrimental trend in JAVELIN Ovarian 100 (HR 1.14).

The test for subgroup differences by combination partner was exploratory and underpowered (nine trials across three subgroups) and was non-significant under both the Wald-type omnibus test (QM = 1.47, df = 2, *p* = 0.48) and the HKSJ-adjusted omnibus test (QM = 0.69, df = 2, 6; *p* = 0.54). Therefore, these subgroup comparisons should be regarded as hypothesis-generating.

### 3.6. Subgroup Analysis by Treatment Setting

By treatment setting, the PFS trends were as follows:

- First-line (*k* = 4): HR 0.85 (95% CI 0.68–1.06 [Wald-type]; HKSJ 0.59–1.24; *I*^2^ = 82.0%).
- Platinum-sensitive recurrent (*k* = 2): HR 0.85 (95% CI 0.74–0.98 [Wald-type], *p* = 0.026; HKSJ 0.55–1.32, *p* = 0.136; *I*^2^ = 0%).
- Platinum-resistant (*k* = 3): HR 0.78 (95% CI 0.67–0.92 [Wald-type], *p* = 0.003; HKSJ 0.58–1.06, *p* = 0.074; *I*^2^ = 36.9%).

The platinum-sensitive recurrent setting showed the most consistent benefit, with both included trials (ATALANTE and ANITA) favoring the addition of ICI and no heterogeneity. In platinum-resistant disease, all three included trials numerically favored the ICI arm (JAVELIN Ovarian 200, HR 0.78; AGO-OVAR 2.29, HR 0.87; KEYNOTE-B96, HR 0.70) with only moderate heterogeneity (*I*^2^ = 36.9%); this estimate should nonetheless be interpreted with caution given the small number of trials.

### 3.7. Overall Survival

OS data were immature in most trials. KEYNOTE-B96 was the only study to show a significant OS gain (median 17.7 vs. 14.0 months; HR 0.82, 95% CI 0.69–0.97; *p* = 0.011) in platinum-resistant disease and the first randomized OS benefit for an ICI-based regimen in ovarian cancer. As this survival result derives from a single trial, it should be regarded as trial-specific evidence rather than a pooled class effect. At the time of the primary analysis, the overall survival (OS) data for DUO-O and FIRST were still immature, rendering a formal pooled OS analysis infeasible. An updated analysis of DUO-O with longer follow-up (53% maturity) subsequently reported a final OS HR of 0.92 (95% CI 0.75–1.11; *p* = 0.378) in the non-tBRCAm ITT population, confirming the absence of a statistically significant OS benefit despite the marked PFS improvement in this triple-combination regimen [34]. Similarly, the final OS analysis of ATALANTE/ENGOT-ov29 (median follow-up 60.1 months; 72.8% maturity) reported a median OS of 35.8 versus 30.6 months favoring the atezolizumab arm (HR 0.83, 95% CI 0.69–0.98; 5-year OS 27% vs. 17%, respectively). However, because both primary endpoints were negative, this survival difference was formally exploratory rather than confirmatory [15].

### 3.8. Safety

Across the primary trials, all-cause grade ≥ 3 adverse event (AE) rates ranged from approximately 69% to 88% in the ICI arms versus 59% to 87% in the control arms (Supplementary Table S6). The direction favored higher grade ≥ 3 toxicity with the ICI-containing regimen in every combination trial, although the absolute increment was modest for the atezolizumab-based regimens (e.g., ATALANTE 88% vs. 87%, ANITA 75% vs. 74%, and AGO-OVAR 2.29 72% vs. 70%) and larger for the chemotherapy- or maintenance-based regimens (e.g., JAVELIN Ovarian 100 72% vs. 63%, DUO-O 72% vs. 62%, and KEYNOTE-B96 83% vs. 71%). The sole exception was the monotherapy trial NINJA (pooled only in SA1), in which single-agent nivolumab produced substantially fewer treatment-related grade 3–4 events than comparator chemotherapy (10.9% vs. 65.2%), consistent with a monotherapy-versus-chemotherapy contrast rather than the addition of an ICI to standard therapy. Among trials using a conventional immune-related adverse event (irAE) definition, irAEs were consistently more frequent in the ICI arms (any-grade irAE approximately 23–40% vs. 0–27%), with grade ≥ 3 irAE rates of approximately 3–13%. Two trials reported on a broader or different basis: IMagyn050 reported an atezolizumab adverse event of special interest rate of 73% vs. 52% (grade ≥ 3 17% vs. 6%), and FIRST reported grade ≥ 2 irAE of 38.4% vs. 13.9%. In NINJA, where the comparator was chemotherapy rather than the same regimen without an ICI, the immune-related category was not higher with nivolumab (50.0% vs. 55.5%). AEs leading to treatment discontinuation were generally higher with ICI, although reporting conventions varied (whole-regimen composite vs. individual-component discontinuation). Treatment-related deaths were uncommon and broadly comparable between arms (no more than approximately 2% per arm in each trial). Because AE definitions, grading, and reporting formats were heterogeneous across trials (treatment-emergent vs. treatment-related AEs; differing irAE grade thresholds and follow-up durations), formal pooled risk ratios were not calculated. Nevertheless, the uniform directional consistency across combination trials supports a high degree of confidence in the conclusion that ICI addition increases grade ≥ 3 toxicity (GRADE: High).

### 3.9. Subgroup Analysis by ICI Agent

Subgroup analysis by ICI agent (primary trials) showed the following pooled PFS HRs: atezolizumab (*k* = 4; HR 0.88, 95% CI 0.81–0.96; *I*^2^ = 0%) and avelumab (*k* = 2; HR 0.96, 95% CI 0.66–1.38; *I*^2^ = 57.1%). Durvalumab (*k* = 1; HR 0.63), dostarlimab (*k* = 1; HR 0.85), and pembrolizumab (*k* = 1; HR 0.70) were each evaluated in a single primary trial. The small number of trials per agent precluded meaningful conclusions regarding agent-specific efficacy (Supplementary Table S5).

### 3.10. Objective Response Rate

Objective response rate (ORR) data were inconsistently reported across trials, with variations in definitions (such as RECIST 1.1 by investigator versus blinded independent central review, BICR) and discrepancies in the reporting of ORRs for the comparison arm. A formal pooled analysis of the ORR was not performed. The descriptive ORR data from the individual trials are summarized in Supplementary Table S4.

### 3.11. Publication Bias

**Figure 5:**
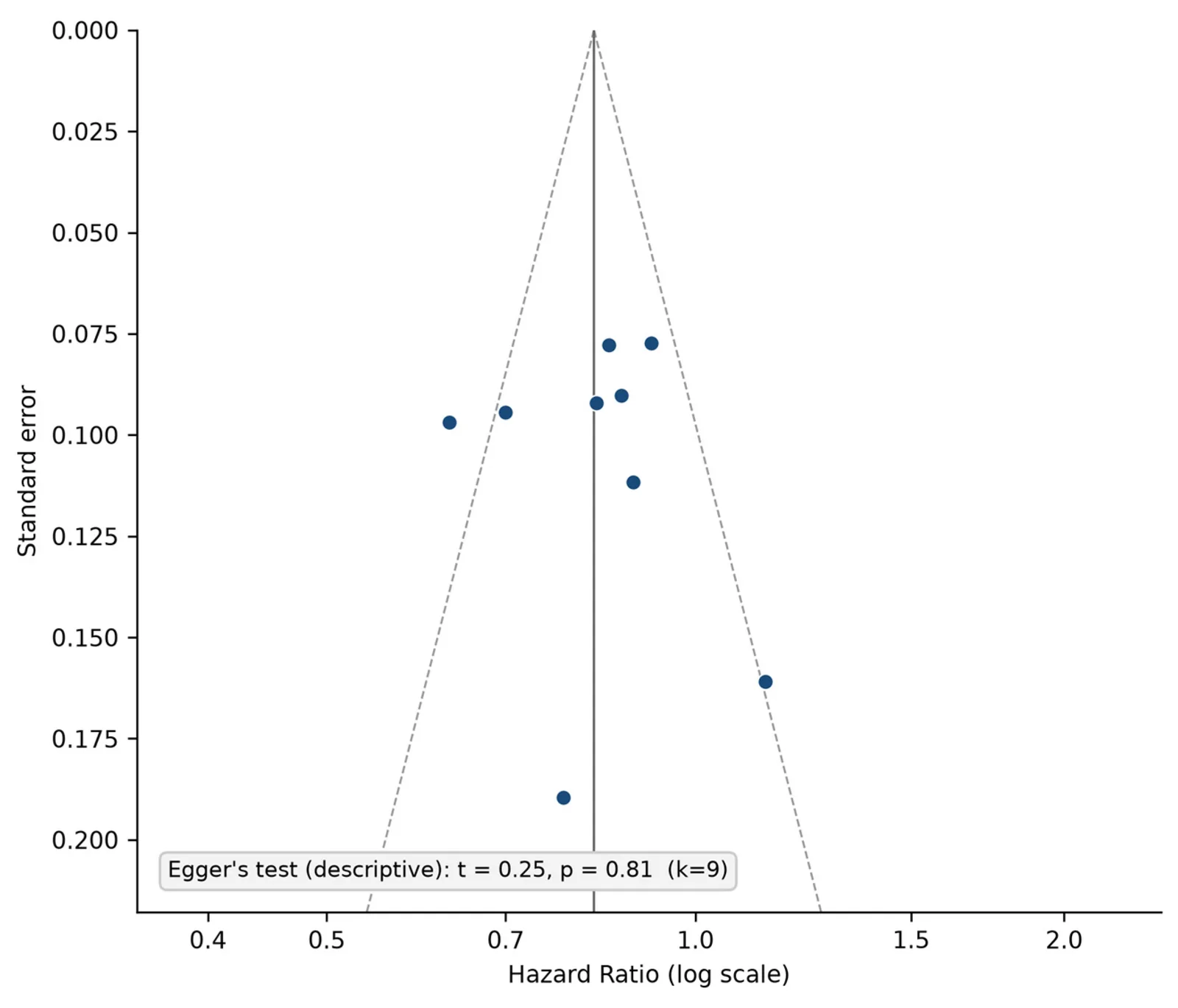
Funnel plot for the assessment of publication bias. Each point represents one trial. Symmetry suggests the absence of publication bias (descriptive Egger’s test, *p* = 0.81; Section 3.11).

Funnel plot inspection showed no apparent asymmetry (Figure 5). Because the primary pool comprised nine trials, the pre-specified threshold for applying Egger’s regression test (≥10 studies) was no longer met, and the test was therefore not applied as a confirmatory analysis. A descriptive Egger test likewise showed no asymmetry (t = 0.25, df = 7, *p* = 0.81); however, with nine studies the test has limited statistical power, and a non-significant result cannot be interpreted as strong evidence that publication bias is absent [25].

### 3.12. Sensitivity Analysis

**Figure 6:**
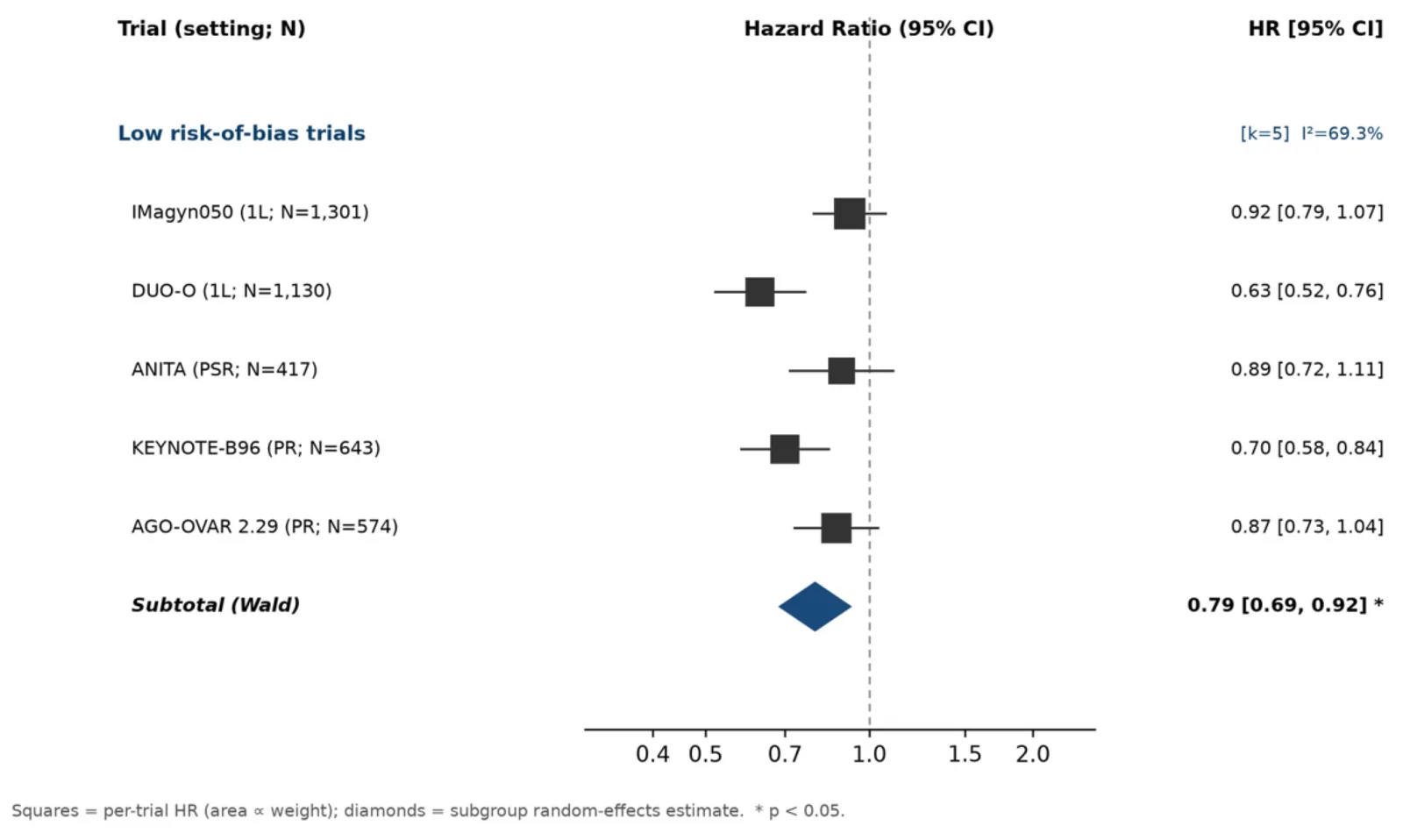
Forest plot of the sensitivity analysis including only low risk-of-bias trials (*k* = 5).

The fixed-effects model yielded a statistically significant pooled HR of 0.83 (95% CI 0.77–0.88), directionally consistent with the random-effects estimate but with narrower confidence intervals, as expected given the assumption of a common effect size (Supplementary Table S3). Excluding the four trials with “some concerns” regarding risk of bias resulted in a pooled HR of 0.79 (95% CI 0.69–0.92; *I*^2^ = 69.3%; *k* = 5) (Figure 6), corroborating the primary finding with a significant pooled estimate. Additional sensitivity analyses assessed the impact of trial selection and classification decisions (Supplementary Table S2). First, inclusion of the NINJA monotherapy trial (SA1; *k* = 10), which did not meet the combination-therapy eligibility criterion, attenuated the overall estimate to non-significance (HR 0.87, 95% CI 0.74–1.03; *I*^2^ = 77.6%) and increased heterogeneity, reflecting the dilution of combination-regimen effects by a monotherapy-versus-chemotherapy contrast with a detrimental effect estimate (HR 1.48). NINJA was pooled solely to benchmark the present estimates against prior meta-analyses that included monotherapy trials, such as that of Vida et al. [10].

Second, the reclassification of DUO-O from the PARP inhibitor + ICI subgroup to the bevacizumab + ICI subgroup, given its triple combination design (durvalumab + bevacizumab + olaparib), shifted subgroup estimates: bevacizumab + ICI (*k* = 5; HR 0.79, 95% CI 0.68–0.90; *I*^2^ = 67.7%) and PARP inhibitor + ICI (*k* = 2; HR 0.86, 95% CI 0.76–0.98; *I*^2^ = 0%). Interestingly, the remaining two PARP + ICI trials (FIRST and ANITA) still showed a significant benefit (HR 0.86, 95% CI 0.76–0.98), suggesting that the PARP–ICI signal is not entirely attributable to the DUO-O triple regimen, although the point estimate increased (from 0.78 to 0.86).

Two additional sensitivity analyses incorporated conference-abstract-only trials. The addition of ATHENA-COMBO and KEYLYNK-001 (SA5) increased the overall pool to *k* = 11 (HR 0.85, 95% CI 0.74–0.99; *I*^2^ = 79.8%) and attenuated the PARP + ICI subgroup to non-significance (*k* = 5; HR 0.85, 95% CI 0.67–1.09; *I*^2^ = 90.1%), driven by the opposing ATHENA-COMBO signal (PFS HR 1.30). Including NRG-GY009 (SA-NRG; *k* = 10) yielded an overall HR of 0.82 (95% CI 0.74–0.91; *I*^2^ = 49.8%) and a bevacizumab + ICI estimate of 0.83 (95% CI 0.75–0.91; *I*^2^ = 29.8%), although NRG-GY009 itself was reported as non-significant under its pre-specified statistical plan.

Third, KEYNOTE-B96 was reclassified from the bevacizumab + ICI subgroup to the chemotherapy + ICI subgroup (Supplementary Table S2). This reclassification was based on the trial’s ±bevacizumab design and the observation that the PFS benefit was as consistent in the non-bevacizumab arm (HR 0.70) as in the bevacizumab arm (HR 0.72). In this SA, the Bev + ICI subgroup (*k* = 3) showed a consistent significant benefit (HR 0.88, 95% CI 0.80–0.97; *I*^2^ = 0%). The Chemo + ICI subgroup (*k* = 3, including KEYNOTE-B96) showed no significant benefit (HR 0.84, 95% CI 0.62–1.13; *I*^2^ = 69.5%). This confirms that the observed benefit in the Bev + ICI subgroup is robust, even when a trial with an uncertain bevacizumab contribution was reclassified.

### 3.13. GRADE Assessment

The certainty of the evidence varied substantially according to the combination partner (Table 3).

- PFS overall: Moderate (downgraded one level for inconsistency; not downgraded for imprecision, as the 95% CI excluded the null).
- PFS with bevacizumab + ICI: Moderate (downgraded for imprecision).
- PFS with PARP inhibitor + ICI: Low (downgraded for inconsistency and imprecision).
- PFS with chemotherapy + ICI: Very low (downgraded for risk of bias, inconsistency, imprecision).
- OS: Very low (immature data).
- Safety (grade ≥ 3 AE increase): High.

Across nine phase III RCTs including 7381 patients, ICI-containing combination therapy was associated with a statistically significant 17% reduction in the hazard of progression or death (HR 0.83, 95% CI 0.73–0.93; *I*^2^ = 55.8%), with a 95% prediction interval (0.62–1.10) whose bounds remained directionally favorable. In exploratory subgroup analyses, bevacizumab-based (HR 0.83, *I*^2^ = 42.9%; GRADE: Moderate) and PARP inhibitor-based combinations (HR 0.78; GRADE: Low) were associated with improved PFS by Wald-type estimation, whereas the two chemotherapy-only (avelumab) trials were not (HR 0.96; GRADE: Very low); the test for subgroup differences was not statistically significant (omnibus Wald QM = 1.47, df = 2, *p* = 0.48; HKSJ-adjusted *p* = 0.54). Because the subgroups comprised only two to four trials, Wald-type and HKSJ intervals were reported in parallel; under the more conservative HKSJ correction, the primary bevacizumab estimate became borderline (95% CI 0.69–1.00), and the PARP inhibitor estimate lost significance (95% CI 0.49–1.24). The bevacizumab signal was nonetheless the more robust of the two; it remained significant under HKSJ when DUO-O was included in the subgroup (*k* = 5; HR 0.79, 95% CI 0.65–0.96, *p* = 0.027), and reassigning KEYNOTE-B96 to the chemotherapy + ICI subgroup left the estimate essentially unchanged (Bev + ICI: *k* = 3, HR 0.88, 95% CI 0.80–0.97, *I*^2^ = 0%).

## 4. Discussion

### 4.1. Comparison with Prior Evidence

Our findings refine those of Vida et al. [10], who reported no meaningful improvement in progression-free survival (PFS) with immune checkpoint inhibitors (ICIs) in ovarian cancer, as reflected by a hazard ratio (HR) of 0.98. That synthesis pooled ICI monotherapy and combination regimens as a single class; accordingly, when we similarly added the monotherapy trial NINJA to the pool (SA1), our overall estimate was likewise attenuated to non-significance (HR 0.87, 95% CI 0.74–1.03). Restricting the synthesis to combination regimens—the pre-specified scope of the present review—yielded a significant PFS benefit, indicating that the previously reported null effect partly reflected dilution by monotherapy contrasts and the absence, at that time, of more recent trials (KEYNOTE-B96 and AGO-OVAR 2.29). This refinement stems from our combination-only scope and pre-specified subgroup analyses, which revealed clinically relevant differences in the robustness of benefit across combination partners. Recent network meta-analyses have also compared immunotherapy-containing regimens for ovarian cancer [35]; however, these relied on indirect cross-regimen comparisons rather than the pre-specified, partner-stratified pairwise synthesis with GRADE-rated certainty that distinguishes the present review. The more modest overall effect size, in turn, is partly explained by the neutral impact of chemotherapy-only combinations, which dilutes the benefits of bevacizumab- and PARP inhibitor–based regimens. Additionally, we incorporated two trials not included in the prior synthesis: KEYNOTE-B96 [11] and AGO-OVAR 2.29 [33], both of which evaluated ICI–bevacizumab combinations in platinum-resistant disease. KEYNOTE-B96 delivered the first significant OS improvement with an ICI in this cancer (HR 0.82, 95% CI 0.69–0.97), a practice-changing result; AGO-OVAR 2.29, although it fell short of significance (PFS HR 0.87), points in the same direction and lends further weight to the rationale for pairing ICIs with bevacizumab-based chemotherapy.

### 4.2. Biological Rationale for Combination-Dependent Efficacy

There is a plausible biological explanation for why partners are important. Bevacizumab normalizes tumor vasculature and dampens immunosuppressive VEGF signaling, potentially nudging the microenvironment from “cold” towards “hot” [8]. This mechanistic hypothesis is consistent with the observed PFS benefit across IMagyn050, ATALANTE, KEYNOTE-B96, and AGO-OVAR 2.29, although none of these trials reported tumor microenvironment endpoints to directly confirm this mechanism. All four trials favored the ICI arm across settings from first-line to platinum-resistant disease, with hazard ratios below 1.0 (effect sizes, 0.70–0.92), consistent with the moderate heterogeneity observed (*I*^2^ = 42.9%).

The role of PARP inhibitors remains unclear. These drugs induce DNA damage, activate the cGAS–STING pathway, and enhance neoantigen presentation, which are mechanisms proposed to augment antitumor immunity [7,36]. However, the considerable heterogeneity within this subgroup (*I*^2^ = 73.8%) necessitated caution. The DUO-O trial, which uniquely combined durvalumab with bevacizumab and olaparib, demonstrated the largest effect, with a hazard ratio of 0.63. When DUO-O was moved into the bevacizumab + ICI subgroup, that subgroup grew to five trials (HR 0.79, 95% CI 0.68–0.90), heterogeneity among the remaining PARP + ICI trials vanished (*I*^2^ = 0%), but their pooled estimate remained significant (HR 0.86, 95% CI 0.76–0.98). While the DUO-O triple regimen amplifies the PARP + ICI signal, the residual benefit among FIRST and ANITA suggests that PARP–ICI synergy extends beyond the triple regimen.

The flat result for chemotherapy-only combinations is also informative. Both trials pairing avelumab with platinum-based chemotherapy or pegylated liposomal doxorubicin (JAVELIN Ovarian 100 and 200) showed no aggregate benefit (HR 0.96, 95% CI 0.66–1.38). One hypothesis for this lack of benefit is that frequent cytotoxic treatments may reduce lymphocyte levels and diminish immune activation [37]; however, the included trials did not report immune cell profiling data to confirm this hypothesis.

### 4.3. Biomarker Considerations (Narrative Synthesis)

Available subgroup data from individual trials indicate that biomarker-guided patient selection can optimize ICI efficacy. In the DUO-O trial, patients with HRD-positive tumors derived substantial benefit (HR 0.49), whereas HR-proficient (HRP) patients also experienced a clinically relevant benefit (HR 0.77) [29], suggesting that ICIs may be particularly valuable in populations in which PARP inhibitors are less effective. However, the predictive value of PD-L1 status has been inconsistent across trials, necessitating the development of novel strategies for immunotherapy in ovarian cancer.

### 4.4. Strengths and Limitations

The strengths of this analysis include: (1) a comprehensive search of four databases identifying 2612 records; (2) incorporation of the most up-to-date trial data, including KEYNOTE-B96 and AGO-OVAR 2.29; (3) a pre-specified subgroup analysis by combination partner, which represents the most clinically actionable comparison; (4) use of GRADE to provide explicit certainty ratings; and (5) extensive sensitivity analyses that confirmed the robustness of the results.

Limitations include: (1) the test for subgroup differences did not reach significance (omnibus Wald QM = 1.47, df = 2, *p* = 0.48; HKSJ-adjusted *p* = 0.54), so all partner-stratified findings must be interpreted as exploratory and hypothesis-generating; subgroup interaction tests are inherently underpowered, typically requiring approximately four times the number of studies to detect a true interaction [23]. The significance of individual subgroup estimates was also method-dependent—both favorable subgroups were significant under Wald-type intervals but attenuated under the more conservative HKSJ correction, reflecting the small number of trials per subgroup—and the number of subgroup and sensitivity analyses performed relative to the number of available trials carries an inherent risk of false-positive findings, which we mitigated by pre-specifying the analysis plan, reporting HKSJ intervals for small-k estimates, and treating all subgroup results as hypothesis-generating; (2) lack of individual patient data, which precluded biomarker-stratified analyses; (3) immature or non-significant OS data in most trials (only KEYNOTE-B96 reached formal OS significance, and this single-trial result should not be generalized as a pooled class effect), which precluded a pooled OS analysis; (4) substantial heterogeneity in the PARP + ICI subgroup, largely driven by the DUO-O trial’s triple regimen (durvalumab + bevacizumab + olaparib), which complicates attribution of benefit specifically to PARP inhibitor–ICI synergy; (5) the ICI monotherapy trial NINJA was excluded from the primary analysis per the combination-therapy scope of the review; its inclusion (SA1) attenuated the overall estimate, and syntheses pooling monotherapy and combination contrasts [10] should be interpreted with this distinction in mind; (6) the ±bevacizumab design of KEYNOTE-B96, although subgroup data suggested consistent benefit irrespective of bevacizumab use; (7) inability to evaluate the role of anti-CTLA-4 agents, which were excluded; and (8) publication-bias assessment relied on visual funnel plot inspection because the pre-specified threshold for Egger’s test (≥10 trials) was not met; with nine trials, the descriptive Egger test (*p* = 0.81) has limited power, and small-study effects cannot be formally excluded.

### 4.5. Clinical Implications

The key implication is that when using an immune checkpoint inhibitor (ICI), the choice of combination partner is more critical than simply deciding to administer an ICI alone. Bevacizumab-containing regimens currently have the strongest supporting evidence among the combination partners (GRADE: Moderate) and should be prioritized in clinical practice, particularly for platinum-resistant disease, as demonstrated in the KEYNOTE-B96 trial. This evidence has begun to reshape practice: in February 2026, the US Food and Drug Administration approved pembrolizumab plus paclitaxel, with or without bevacizumab, for PD-L1-positive (CPS ≥ 1) platinum-resistant epithelial ovarian, fallopian tube, or primary peritoneal cancer based on KEYNOTE-B96, the first immune checkpoint inhibitor indication in ovarian cancer [38]. The 2026 NCCN guidelines list this regimen as an option for PD-L1-positive platinum-resistant disease [39]. PARP inhibitor-based combinations appear promising, although the certainty of evidence is low. In contrast, chemotherapy-only combinations cannot be recommended based on currently available data.

### 4.6. Future Directions

Future research should prioritize: (1) finding biomarkers beyond PD-L1 that can predict which patients will benefit most; (2) understanding the distinct and combined roles of each drug in multi-drug therapies, possibly using component network meta-analyses, such as for the DUO-O triple regimen; (3) exploring new combinations of immune checkpoint inhibitors, such as anti-TIGIT and anti-LAG-3, specifically for ovarian cancer; and (4) obtaining comprehensive overall survival data from ongoing trials, especially the FIRST trial.

## 5. Conclusions

Taken together, these nine phase III RCTs indicate that ICI-containing combination therapy improves PFS in advanced ovarian cancer (HR 0.83, 95% CI 0.73–0.93; GRADE: Moderate certainty), in contrast to earlier syntheses that pooled monotherapy and combination regimens and reported null overall effects. The benefit pattern appears partner-specific, although the test for subgroup differences was not statistically significant and these analyses remain exploratory and hypothesis-generating: bevacizumab-based combinations showed the most robust PFS benefit (GRADE: Moderate), PARP inhibitor–based combinations were favorable but heavily dependent on the DUO-O triple regimen and of low certainty, and chemotherapy-only (avelumab) combinations showed no benefit (GRADE: Very low). KEYNOTE-B96 provides the first randomized OS signal for an ICI regimen in this disease, albeit from a single trial. Collectively, these data support a partner-specific, biomarker-guided strategy for integrating immunotherapy into ovarian cancer treatment.

## Figures and Tables

**Table 1:** Characteristics of included studies.

Trial	Year	Setting	*N*	ICI Agent	Combination Partner	Design	PFS HR (95% CI)
IMagyn050	2021	First-line	1301	Atezolizumab	Chemo + Bevacizumab	DB, PC	0.92 (0.79–1.07)
JAVELIN Ovarian 100	2021	First-line	998	Avelumab	Chemo → Maintenance	OL	1.14 (0.83–1.56)
DUO-O	2026	First-line	1130	Durvalumab	Chemo + Bev → Bev + Olaparib maint	DB, PC	0.63 (0.52–0.76)
FIRST	2025	First-line	1138	Dostarlimab	Chemo → Niraparib maint	DB, PC	0.85 (0.73–0.99)
ATALANTE	2023	PSR	614	Atezolizumab	Chemo + Bevacizumab	OL	0.83 (0.69–0.99)
ANITA	2024	PSR	417	Atezolizumab	Atezo + Platinum → Atezo + Niraparib maint	DB, PC	0.89 (0.71–1.10)
JAVELIN Ovarian 200	2021	PR	566	Avelumab	±PLD	OL	0.78 (0.59–1.24) †
KEYNOTE-B96	2026	PR	643	Pembrolizumab	Paclitaxel ± Bevacizumab	DB, PC	0.70 (0.58–0.84)
AGO-OVAR 2.29	2026	PR	574	Atezolizumab	Bev + non-Pt Chemo	DB, PC	0.87 (0.73–1.04)

DB, double-blind; PC, placebo-controlled; OL, open-label; PSR, platinum-sensitive recurrent; PR, platinum-resistant; Chemo, chemotherapy; Bev, bevacizumab; maint, maintenance; PLD, pegylated liposomal doxorubicin. JAVELIN Ovarian 100: combination arm HR reported (chemotherapy plus avelumab followed by avelumab maintenance vs. control; open-label design). JAVELIN Ovarian 200: avelumab + PLD vs. PLD arm HR reported. NINJA compared ICI monotherapy with chemotherapy and did not meet the combination-therapy eligibility criterion; it was pooled only in sensitivity analysis SA1 (Section 2.6 and Section 3.12). ANITA was classified as PARP+ICI: atezolizumab was combined with platinum chemotherapy, followed by atezolizumab + niraparib maintenance, classified by maintenance phase, as the majority of PFS events occurred during maintenance therapy. DUO-O classified as PARP+ICI in primary analysis: triple combination (durvalumab + bevacizumab + olaparib); reclassified to Bev+ICI in sensitivity analysis SA2. † JAVELIN Ovarian 200: reported as repeated 93.1% CI (alpha-spending adjusted); SE was back-calculated from the reported CI bounds for meta-analytic pooling.

**Table 2:** Risk of bias assessment (RoB 2.0).

Trial	D1: Randomization	D2: Deviations	D3: Missing Data	D4: Measurement	D5: Reporting	Overall
IMagyn050	Low	Low	Low	Low	Low	Low
JAVELIN Ovarian 100	Low	Some concerns	Low	Low	Some concerns	Some concerns
DUO-O	Low	Low	Low	Low	Low	Low
FIRST	Low	Low	Low	Low	Some concerns	Some concerns
ATALANTE	Low	Some concerns	Low	Low	Low	Some concerns
ANITA	Low	Low	Low	Low	Low	Low
JAVELIN Ovarian 200	Low	Some concerns	Low	Some concerns	Low	Some concerns
KEYNOTE-B96	Low	Low	Low	Low	Low	Low
AGO-OVAR 2.29	Low	Low	Low	Low	Low	Low

D1 = Randomization process; D2 = Deviations from intended interventions; D3 = Missing outcome data; D4 = Measurement of the outcome; D5 = Selection of the reported result. Concerns: JAVELIN Ovarian 100 (open-label design [D2], early termination for futility [D5]), FIRST (interim analysis for PFS [D5]), ATALANTE (partially open-label [D2]), and JAVELIN Ovarian 200 (open-label [D2], investigator-assessed PFS without BICR [D4]). The monotherapy trial NINJA (pooled only in SA1) was rated as having some concerns (open-label [D2]; investigator-assessed PFS without BICR [D4]). Overall (nine primary trials): 5 Low (56%), 4 Some concerns (44%), 0 High risk.

**Table 3:** GRADE summary of findings.

Outcome	*k*	Pooled HR (95% CI)	*I* ^2^	GRADE	Reason for Downgrading
PFS (overall)	9	0.83 (0.73–0.93)	55.8%	⊕⊕⊕◯ Moderate	Inconsistency (one level; 95% CI excludes the null)
PFS (Bev + ICI)	4	0.83 (0.74–0.93)	42.9%	⊕⊕⊕◯ Moderate	Imprecision (HKSJ CI includes null)
PFS (PARP + ICI)	3	0.78 (0.63–0.96)	73.8%	⊕⊕◯◯ Low	Inconsistency, imprecision
PFS (Chemo + ICI)	2	0.96 (0.66–1.38)	57.1%	⊕◯◯◯ Very low	Risk of bias (2 open-label; 1 without BICR), inconsistency, imprecision
OS	—	—	—	⊕◯◯◯ Very low	Immature data (only KEYNOTE-B96 positive)
Safety (grade ≥ 3 AE)	—	—	—	⊕⊕⊕⊕ High	Consistent across trials

GRADE = Grading of Recommendations, Assessment, Development, and Evaluations. Starting point: RCT = High. Downgraded for risk of bias, inconsistency (*I*^2^ > 50% or direction disagreement), imprecision (95% CI includes null or very wide), indirectness, and publication bias. PFS overall: downgraded one level for inconsistency (*I*^2^ = 55.8%); not downgraded for imprecision (95% CI 0.73–0.93 excludes the null), risk of bias (five of nine trials at low risk; the low-risk-only sensitivity analysis was consistent), or publication bias (funnel plot symmetric; small-study effects cannot be formally excluded at *k* = 9). PFS Bev+ICI: downgraded one level to moderate for imprecision because the primary *k* = 4 HKSJ-adjusted interval included the null (0.69–1.00, *p* = 0.051) despite a Wald-type interval excluding 1.0 (0.74–0.93). It was not downgraded further given the low heterogeneity (*I*^2^ = 42.9%), consistent direction across all four trials (HR 0.70–0.92), and retention of significance under HKSJ once DUO-O was included (SA2, *k* = 5; HR 0.79, 95% CI 0.65–0.96, *p* = 0.027). However, the inclusion of DUO-O in the sensitivity analysis involved reclassification of a triple combination regimen and was therefore regarded as a secondary, confirmatory finding rather than overriding the imprecision assessment of the primary subgroup definition. PFS PARP+ICI: downgraded for inconsistency (*I*^2^ = 73.8%), driven by the DUO-O triple regimen (HR 0.63 vs. FIRST 0.85 and ANITA 0.89) and imprecision (HKSJ CI includes null). The Wald-type significance (0.63–0.96) was not retained under HKSJ (0.49–1.24) and was lost when abstract-only trials were added (SA5), supporting a low rating. PFS Chemo+ICI: downgraded 3 levels—both trials have “some concerns” RoB (open-label; JAVELIN Ovarian 200 without BICR); *I*^2^ = 57.1% with opposing directions (JAVELIN 200 HR 0.78 vs. JAVELIN 100 HR 1.14); very wide CI (0.66–1.38) based on only two trials. GRADE certainty symbols: ⊕⊕⊕⊕ = high; ⊕⊕⊕◯ = moderate; ⊕⊕◯◯ = low; ⊕◯◯◯ = very low certainty of evidence.

## Data Availability

All data were obtained from published trials. The R analysis code and extracted data are available from the corresponding author upon reasonable request.

## Supplementary Materials

The following supporting information can be downloaded at: https://www.mdpi.com/article/10.3390/cancers18182971/s1, Table S1: Search strategy for PubMed, Embase, CENTRAL, and Web of Science. Table S2: Sensitivity analyses of trial selection and classification decisions. Table S3: Random-effects vs. fixed-effect estimates and heterogeneity statistics. Table S4: Descriptive objective response rates (ORR) by trial. Table S5: PFS subgroup analysis by ICI agent. Table S6: Quantitative safety outcomes by trial. Table S7: GRADE evidence profile (per-outcome certainty ratings and reasons for downgrading). Table S8: Combination-partner classification rule and trial-by-trial assignment rationale; PRISMA 2020 checklist.
