# Supplementary material for: Immune Checkpoint Inhibitor Combinations in Advanced Ovarian Cancer: A Systematic Review and Meta-Analysis of Randomized Controlled Trials
Source: Cancers (Basel). 2026 Sep 14;18(18):2971. doi: 10.3390/cancers18182971 (PMC13605854; doi:10.3390/cancers18182971)
Supplement: Supplementary file 1 [file cancers-18-02971-s001.zip › cancers-4527607-supplementary.pdf]

Supplementary Materials

Immune Checkpoint Inhibitor Combinations in Advanced Ovarian Cancer:
A Systematic Review and Meta-Analysis of Randomized Controlled Trials

Supplementary Table S1. Search Strategy

Databases searched from inception to March 1, 2026, include PubMed/MEDLINE, Embase (via Ovid), Cochrane Central Register of Controlled Trials (CENTRAL), and Web of Science (Science Citation Index). Conference proceedings (ASCO, ESMO, and SGO; 2018–2026) were manually searched. The PubMed/MEDLINE strategy is shown below; equivalent syntax-adapted strategies were applied to the other databases.

Set	Concept	Search terms (PubMed/MEDLINE)
#1	Population	"Ovarian Neoplasms"[Mesh] OR "Fallopian Tube Neoplasms"[Mesh] OR "Peritoneal Neoplasms"[Mesh] OR ((ovar[tiab] OR "fallopian tube"[tiab] OR peritone[tiab]) AND (cancer[tiab] OR carcinoma[tiab] OR neoplasm[tiab] OR tumour[tiab] OR tumor[tiab] OR malignan[tiab]))
#2	Immune checkpoint inhibitor	"Immune Checkpoint Inhibitors"[Mesh] OR "Programmed Cell Death 1 Receptor"[Mesh] OR "B7-H1 Antigen"[Mesh] OR PD-1[tiab] OR PD-L1[tiab] OR "PD 1"[tiab] OR "PD L1"[tiab] OR "checkpoint inhibitor*"[tiab] OR pembrolizumab[tiab] OR nivolumab[tiab] OR atezolizumab[tiab] OR avelumab[tiab] OR durvalumab[tiab] OR dostarlimab[tiab]
#3	Combination partner	"Poly(ADP-ribose) Polymerase Inhibitors"[Mesh] OR PARP[tiab] OR olaparib[tiab] OR niraparib[tiab] OR

		rucaparib[tiab] OR “Bevacizumab”[Mesh] OR bevacizumab[tiab] OR “Antineoplastic Combined Chemotherapy Protocols”[Mesh] OR chemotherapy[tiab] OR carboplatin[tiab] OR cisplatin[tiab] OR paclitaxel[tiab] OR doxorubicin[tiab] OR gemcitabine[tiab]
#4	Study design filter	“Randomized Controlled Trial”[Publication Type] OR “Controlled Clinical Trial”[Publication Type] OR randomized[tiab] OR randomised[tiab] OR randomly[tiab] OR “controlled trial”[tiab] OR RCT[tiab] OR “phase 3”[tiab] OR “phase III”[tiab]
#5	Combination	#1 AND #2 AND #3 AND #4

MeSH = Medical Subject Headings; [tiab] = title/abstract field; [Mesh] = MeSH term. The Embase strategy used Emtree equivalents (e.g., exp ovary tumor/, exp immune checkpoint inhibitor/), and CENTRAL and Web of Science used analogous free-text term sets. No language or date restrictions were applied at the database level of the review. The per-database record counts and full PRISMA 2020 selection counts are reported in a flow diagram (Figure 1).

Supplementary Table S2. Sensitivity Analyses of Trial Selection and Classification Decisions

Analysis	Scope	k	Pooled HR (95% CI)	I²	Interpretation
Primary analysis	All combination-therapy trials	9	0.83 (0.73–0.93)	55.8%	Significant (HKSJ p = 0.007)
Fixed-effects	All trials	9	0.83 (0.77–0.88)	55.8%	Significant; narrower CI
SA-RoB	Low risk of bias only	5	0.79 (0.69–0.92)	69.3%	Significant (Wald; HKSJ 0.65–0.98)
SA1: Add NINJA	Overall	10	0.87 (0.74–1.03)	77.6%	Non-significant; heterogeneity increased
SA2: DUO-O → Bev+ICI	Bev + ICI	5	0.79 (0.68–0.90)	67.7%	Significant (Wald; HKSJ 0.67–0.96)
SA2 subgroup	PARP + ICI	2	0.86 (0.76–0.98)	0%	Significant; attenuated (from 0.78)
SA2 subgroup	Chemo + ICI	2	0.96 (0.66–1.38)	57.1%	Unchanged
SA3: KEYNOTE-B96 → Chemo+ICI	Bev + ICI	3	0.88 (0.80–0.97)	0%	Significant; robust
SA3 subgroup	Chemo + ICI	3	0.84 (0.62–1.13)	69.5%	Non-significant
SA5: + Abstract-only trials	Overall	11	0.85 (0.74–0.99)	79.8%	Estimate attenuated; high heterogeneity
SA5 subgroup	PARP + ICI	5	0.85 (0.67–1.09)	90.1%	Non-significant with abstract-only trials
SA-NRG: + NRG-GY009	Overall	10	0.82 (0.74–0.91)	49.8%	Significant; reduced heterogeneity
SA-NRG subgroup	Bev + ICI	5	0.83 (0.75–0.91)	29.8%	Robust; reduced heterogeneity

SA: Sensitivity Analysis. Overall and subgroup estimates with k ≥ 6 used REML with Hartung-Knapp-Sidik-Jonkman (HKSJ) adjustment. For subgroup and sensitivity estimates with k < 6, Wald-type CIs were reported as the primary estimate, with HKSJ intervals provided alongside the key subgroups; the two methods can diverge appreciably at small k values [21]. SA-RoB: Trials

with “some concerns” regarding risk of bias were excluded. SA1: NINJA added (ICI monotherapy vs. chemotherapy; does not meet the combination eligibility criterion; pooled only here to benchmark against prior meta-analyses including monotherapy trials [10]). SA2: DUO-O reclassified from PARP+ICI to Bev+ICI (triple combination including bevacizumab). SA3: KEYNOTE-B96 reclassified from Bev+ICI to Chemo+ICI (±bevacizumab factorial design). Bev + ICI (k = 3): HR 0.88 (95% CI 0.80–0.97; I^2 = 0%); Chemo + ICI (k = 3, including KEYNOTE-B96): HR 0.84 (95% CI 0.62–1.13; I^2 = 69.5%). SA-NRG: NRG-GY009 added (k = 10; overall HR 0.82, 95% CI 0.74–0.91; Bev + ICI k = 5: HR 0.83, 95% CI 0.75–0.91; I^2 = 29.8%). SA5: ATHENA-COMBO [16,17] and KEYLYNK-001 [24] added (conference abstracts only; eligible for the review but excluded from the primary analysis; included here to test the robustness of the primary k = 9 analysis). Overall (k = 11): HR 0.85 (95% CI 0.74–0.99; I^2 = 79.8%). ATHENA-COMBO contributed PFS HR 1.30 (rucaparib + nivolumab vs. rucaparib; PARP+ICI harm), and KEYLYNK-001 contributed ITT PFS HR 0.71 (0.61–0.84; pembrolizumab + olaparib vs. control). Even with the favorable KEYLYNK-001 estimate, adding the two abstract-only trials attenuated the PARP+ICI subgroup to non-significance (k = 5; HR 0.85, 95% CI 0.67–1.09; I^2 = 90.1%), driven by the opposing ATHENA-COMBO results.

Supplementary Table S3. Random-effects vs. Fixed-effect Estimates and Heterogeneity Statistics

Analysis	k	Random-effects HR (95% CI)	Fixed-effect HR (95% CI)	I^2	τ^2	Cochran's Q (p)
Overall PFS	9	0.83 (0.73–0.93) †	0.83 (0.77–0.88)	55.8%	0.012	17.85 (p = 0.022)
Bev + ICI	4	0.83 (0.74–0.93)	0.84 (0.77–0.91)	42.9%	0.006	5.26 (p = 0.15)
PARP + ICI	3	0.78 (0.63–0.96)	0.78 (0.71–0.87)	73.8%	0.025	7.47 (p = 0.024)
Chemo + ICI	2	0.96 (0.66–1.38)	0.97 (0.76–1.24)	57.1%	0.041	2.33 (p = 0.13)

Random-effects estimator: REML. † The overall estimate used the Hartung-Knapp-Sidik-Jonkman (HKSJ) adjustment ($k \geq 6$); subgroup random-effects CIs ($k < 6$) were Wald-type, consistent with Section 2.6. The fixed-effect (inverse-variance) model yielded directionally concordant but narrower intervals and reached significance overall (HR 0.83, $p < 0.001$), reflecting its assumption of a single common effect; the random-effects model was retained as the primary analysis given the between-trial heterogeneity (overall I^2 = 55.8%). τ^2 = between-study variance; Q = Cochran's heterogeneity statistic.

Supplementary Table S4. Descriptive Objective Response Rate (ORR) by Trial

Trial	Setting	Response population; assessment	ORR, ICI arm	ORR, control arm
IMagyn050	1L	Measurable residual disease (n = 251 / 239); RECIST 1.1 (investigator)	93% (233/251) ‡	89% (212/239)
JAVELIN Ovarian 100	1L	All randomized (n = 331 / 335); RECIST 1.1 (BICR)	36% (119/331) §	30% (102/335)
DUO-O	1L	Not a reported endpoint	NR ¶	NR ¶
FIRST	1L	Measurable disease at baseline (n = 561 / 284); RECIST 1.1 (investigator)	88.4% (496/561) §	88.0% (250/284)
ATALANTE	PSR	ITT; RECIST 1.1 (investigator)	62%	66%
JAVELIN Ovarian 200	PR	ITT; RECIST 1.1 (BICR)	13% (25/188)	4% (8/190)
NINJA	PR	Response-evaluable (measurable disease); RECIST 1.1 (investigator)	7.6% (9/119)	13.2% (15/114)
KEYNOTE-B96	PR	Response-evaluable; RECIST 1.1 (investigator)	50% (139/276)	41% (116/282)
AGO-OVAR 2.29	PR	Measurable disease (n = 503); RECIST 1.1 (investigator)	40% (101/255)	44% (108/248)

ORR values are shown for the ICI-containing (experimental) and control arms, as reported in each source. ‡ IMagyn050: ORR is from the primary publication [27]; the response-evaluable population comprised patients with a measurable residual disease after primary surgery. In the PD-L1–positive population, the ORR was 92% (156/169) and 90% (142/158). § JAVELIN Ovarian 100: per-arm ORR is from the trial appendix (Supplementary Table 1, blinded independent central review) [28]; values are for the concurrent chemotherapy + avelumab →

avelumab maintenance arm (the combination contrast used in this meta-analysis) vs. chemotherapy → observation control among all randomized patients (complete response rates 10% vs. 7%). By investigator assessment, the corresponding rates were 31% (103/331) vs. 28% (93/335); the avelumab-maintenance-only arm had a BICR ORR of 30% (101/332). FIRST: ORR was from Supplementary Table S4 of the primary publication [30], among patients with measurable disease at baseline (arm 3 dostarlimab + niraparib vs. arm 2 niraparib; complete response rates 43.5% vs. 46.5%). ¶ DUO-O: the primary publication [29] reports progression-free and overall survival but does not report an objective response rate (a first-line, maintenance-inclusive design without a formal ORR endpoint); therefore, ORR is not reported. JAVELIN Ovarian 200: values are for the avelumab + pegylated liposomal doxorubicin combination arm (the ICI combination contrast used in this meta-analysis); the avelumab monotherapy arm ORR was 4% (7/188 patients). KEYNOTE-B96: Overall population ORR is shown; in the PD-L1 CPS ≥ 1 population, ORR was 53% (107/202) vs. 47% (95/204). NR = not reported in the source available for extraction; The differing response populations (ITT vs. measurable disease subset), assessment methods (investigator vs. BICR), and incomplete reporting precluded formal pooling, as noted in Section 3.10 of this paper. NINJA is the ICI monotherapy trial; it is not eligible for the primary analysis and is shown here for completeness (pooled only in SA1 as a benchmark; Supplementary Table S2).

Supplementary Table S5. PFS Subgroup Analysis by ICI Agent

ICI agent	k	Trials	Pooled PFS HR (95% CI)	I^2
Atezolizumab	4	IMagyn050, ATALANTE, ANITA, AGO-OVAR 2.29	0.88 (0.81–0.96)	0%
Avelumab	2	JAVELIN Ovarian 100, JAVELIN Ovarian 200	0.96 (0.66–1.38)	57.1%
Durvalumab	1	DUO-O	0.63 (0.52–0.76)	—
Dostarlimab	1	FIRST	0.85 (0.73–0.99)	—
Pembrolizumab	1	KEYNOTE-B96	0.70 (0.58–0.84)	—

Multi-trial subgroups ($k < 6$) used Wald-type random-effects confidence intervals (CIs) (REML). Single-trial agents showed individual trial HR (95% CI). The small number of trials per agent and the confounding of ICI agents with combination partners and treatment settings preclude conclusions regarding agent-specific efficacy (Section 3.9).

Supplementary Table S6. Quantitative Safety Outcomes by Trial

Trial	Safety N (ICI / control)	Grade ≥3 AE, % (ICI vs. control)	irAE, % (ICI vs. control)	AE → discontinuation, % (ICI vs. control)	Treatment-related deaths, n (ICI vs. control)
IMagyn050	642 / 644	79 vs. 73	73 vs. 52 [j]	26 vs. 22	4 (1%) vs. 5 (1%)
JAVELIN Ovarian 100	329 / 334	72 vs. 63	28 vs. 0 [b]	19 vs. 7	1 (<1%) vs. 0
DUO-O	378 / 376	72 vs. 62 [d]	31 vs. 11 [b]	18 vs. 7 [e]	1 (AML) [i] vs. 0
FIRST	745 / 417	85.6 vs. 81.8	38.4 vs. 13.9 [c]	41.5 vs. 34.3	3 (0.4%) vs. 2 (0.5%)
ATALANTE	408 / 201	88 vs. 87	27 vs. 15 [b]	25 vs. 18 [e]	3 vs. 2 [f]
ANITA	207 / 209	75 vs. 74	23 vs. 9 [b]	NR [g]	2 vs. 0
JAVELIN Ovarian 200	182 / 177	69 vs. 59	28 vs. 5 [b]	4 vs. 7 [a]	0 vs. 1 (1%)
NINJA	156 / 155	10.9 vs. 65.2 [a]	50.0 vs. 55.5 [b]	7 vs. 10	0 vs. 0
KEYNOTE-B96	320 / 318	83 vs. 71	39 vs. 19 [b]	43 vs. 34	4 (1%) vs. 5 (2%)
AGO-OVAR 2.29	281 / 286	72 vs. 70	40 vs. 27 [b]	16 vs. 14 [e]	6 vs. 6 [h]

Values are percentages of the safety analysis set unless otherwise stated; ICI = ICI-containing (experimental) arm. Grade ≥3 AE are all-cause (treatment-emergent) rates unless otherwise specified. irAE = immune-related adverse events. Reporting definitions differ across trials, which — as stated in Section 3.8 — precluded the calculation of pooled risk ratios. Footnotes: [a] Treatment-related (rather than all-cause) figure (for these trials, an all-cause grade ≥3 rate was not separately reported, or the treatment-related discontinuation rate is shown). [b] any-grade irAE. [c] grade ≥2 treatment-emergent irAE (FIRST reporting convention). [d] DUO-O values are for the durvalumab + olaparib arm vs. control — the arm contributing HR 0.63 to the primary pooled analysis — accumulated over the whole treatment course (chemotherapy + maintenance) from the 18 September 2023 data cut-off [29]; the grade ≥3 AE rate shown is all-cause (the durvalumab-only arm was 66% vs. 62%), and irAE is the any-grade immune-mediated rate. [e] Discontinuation of the ICI/placebo component specifically (a single whole-regimen composite was not reported). [f] ATALANTE reported five treatment-related deaths, three in the atezolizumab arm and two in the placebo arm. [g] ANITA reported discontinuation only by treatment phase (maintenance niraparib and maintenance ICI/placebo separately), not as a single composite. [h] therapy-related deaths, as per the CONSORT diagram. NR = not reported in the source available for extraction. For trials reporting both, the treatment-related grade ≥3 AE rates were as follows: IMagyn050 75 vs. 67; JAVELIN Ovarian 100 62 vs. 56; FIRST 76.6 vs. 72.7; JAVELIN Ovarian 200 43 vs. 32; KEYNOTE-B96 68 vs. 55; ATALANTE 33 vs. 35; ANITA 65 vs. 63; AGO-OVAR 2.29 (related to atezolizumab/placebo) 23 vs. 12. [i] DUO-O: one death (acute myeloid leukemia) was attributed to olaparib in the durvalumab + olaparib arm and none to durvalumab; AEs with a fatal outcome, regardless of causality, occurred in 2.1% vs. 1.1% (ICI vs. control). [j] IMagyn050: the grade ≥3

AE shown is all-cause (79% vs. 73%); the immune-related figure is the atezolizumab adverse-event-of-special-interest (AESI) rate (any grade; grade ≥3 was 17% vs. 6%), with discrete terms including rash 41% vs. 26% and hypothyroidism 26% vs. 13%; the death column shows treatment-related grade 5 events (4 vs. 5), while AEs with a fatal outcome regardless of causality occurred in 1% vs. 1% [27].

Supplementary Table S7. GRADE Evidence Profile

GRADE = Grading of Recommendations, Assessment, Development, and Evaluations. Starting point: RCT = High. Downgraded for risk of bias, inconsistency (I^2 >50% or direction disagreement), imprecision (95% CI includes null or very wide), indirectness, and publication bias. PFS overall: downgraded one level for inconsistency (I^2 = 55.8%); not downgraded for imprecision (95% CI 0.73–0.93 excludes the null), risk of bias (five of nine trials at low risk; the low-risk-only sensitivity analysis was consistent), or publication bias (funnel plot symmetric; small-study effects cannot be formally excluded at k = 9). PFS Bev+ICI: downgraded one level to moderate for imprecision because the primary k = 4 HKSJ-adjusted interval included the null (0.69–1.00, p = 0.051) despite a Wald-type interval excluding 1.0 (0.74–0.93). It was not downgraded further given the low heterogeneity (I^2 = 42.9%), consistent direction across all four trials (HR 0.70–0.92), and retention of significance under HKSJ once DUO-O was included (SA2, k = 5; HR 0.79, 95% CI 0.65–0.96, p = 0.027). However, the inclusion of DUO-O in the sensitivity analysis involved reclassification of a triple combination regimen and was therefore regarded as a secondary, confirmatory finding rather than overriding the imprecision assessment of the primary subgroup definition. PFS PARP+ICI: downgraded for inconsistency (I^2 = 73.8%), driven by the DUO-O triple regimen (HR 0.63 vs. FIRST 0.85 and ANITA 0.89) and imprecision (HKSJ CI includes null). The Wald-type significance (0.63–0.96) was not retained under HKSJ (0.49–1.24) and was lost when abstract-only trials were added (SA5), supporting a low rating. PFS Chemo+ICI: downgraded 3 levels — both trials have "some concerns" RoB (open-label; JAVELIN Ovarian 200 without BICR); I^2 = 57.1% with opposing directions (JAVELIN 200 HR 0.78 vs. JAVELIN 100 HR 1.14); very wide CI (0.66–1.38) based on only two trials.).

Outcome	k	Pooled HR (95% CI)	I^2	Certainty	Reason(s) for downgrading
PFS (overall)	9	0.83 (0.73–0.93)	55.8%	⊕⊕⊕◯ Moderate	Inconsistency (one level); not downgraded for imprecision (CI excludes the null), risk of bias (five of nine trials at low risk; low-RoB sensitivity analysis consistent), or publication bias
PFS (Bev + ICI)	4	0.83 (0.74–0.93)	42.9%	⊕⊕⊕◯ Moderate	Imprecision (HKSJ CI 0.69–1.00, p = 0.051);

					consistent direction across all four trials (HR 0.70–0.92); significant under HKSJ once DUO-O included (SA2, k = 5)
PFS (PARP + ICI)	3	0.78 (0.63–0.96)	73.8%	⊕⊕◯◯ Low	Inconsistency (DUO-O 0.63 vs. FIRST 0.85 and ANITA 0.89); imprecision (HKSJ CI includes the null; signal lost when abstract-only trials added, SA5)
PFS (Chemo + ICI)	2	0.96 (0.66–1.38)	57.1%	⊕◯◯◯ Very low	Risk of bias (both open-label; JAVELIN Ovarian 200 without BICR); inconsistency (opposing directions); imprecision (k = 2, wide CI)
OS	—	—	—	⊕◯◯◯ Very low	Immature data; single positive trial (KEYNOTE-B96)
Safety (grade ≥3 AE)	—	—	—	⊕⊕⊕⊕ High	Consistent across trials; no domains downgraded

Supplementary Table S8. Combination-Partner Classification Rule and Trial-by-Trial Assignment Rationale

Because multi-agent regimens can contain more than one partner (e.g., durvalumab + bevacizumab + olaparib in DUO-O), categories are not mutually exclusive at the regimen level; the primary assignment and its rationale are documented for each trial in Supplementary Table S8, and alternative assignments were tested in pre-specified sensitivity analyses (SA2 and SA3).

Level	Rule	Trials assigned	Notes
1	ICI-specific randomization in the maintenance phase: the maintenance partner defines the subgroup	ANITA (niraparib); DUO-O (olaparib)	DUO-O reclassified to Bev + ICI in SA2 given its durvalumab + bevacizumab + olaparib triple regimen
2	Otherwise, a concomitant non-chemotherapy backbone agent co-administered with the ICI defines the subgroup	IMagyn050, ATALANTE, AGO-OVAR 2.29, KEYNOTE-B96 (bevacizumab)	KEYNOTE-B96: ±bevacizumab factorial design with bevacizumab permitted in both arms; assignment varied in SA3
3	Regimens adding an ICI to chemotherapy alone	JAVELIN Ovarian 100; JAVELIN Ovarian 200	No non-chemotherapy combination partner
—	ICI monotherapy (no combination partner)	NINJA — not eligible for the primary analysis	Pooled only in SA1 to benchmark against prior meta-analyses that included monotherapy trials [10]
